# The Impact of Night Work on the Sleep and Health of Medical Staff—A Review of the Latest Scientific Reports

**DOI:** 10.3390/jcm13154505

**Published:** 2024-08-01

**Authors:** Katarzyna Czyż-Szypenbejl, Wioletta Mędrzycka-Dąbrowska

**Affiliations:** Department of Anaesthesiology Nursing & Inte and Intensive Care, Faculty of Health Sciences, Medical University of Gdansk, 80-211 Gdansk, Poland; katarzyna.czyz-szypenbejl@gumed.edu.pl

**Keywords:** nurse, healthcare workers, night work, sleep, sleep disorders, health

## Abstract

**Introduction:** Employees working in shifts are exposed to many threats affecting their health, quality of life and safety at work. Those who perform their work only at night are particularly vulnerable. The purpose of the review is to identify risks to the health, quality of life and sleep of shift health workers. **Method:** A systematic review (SR) was used in the analysis. Electronic databases were searched. The search was limited to the latest studies published in the last five years: 2019–2023. **Results:** Finally, 36 articles were included in the review. Most authors have shown a link between sleep disturbance or its quality and shift work/night work. Moreover, a three-shift schedule was the most significant factor for poorer subjective sleep quality when compared to other work schedules. Furthermore, many authors have shown a link between shift/night work and health problems, which include cardiometabolic risk, glucose intolerance, breast cancer and immune vulnerability. **Conclusions:** The research results clearly show a significant impact of night work on the increased risk of sleep disorders and health disturbance. Healthcare workers should be aware of the risks associated with night work in order to take measures preventing sleep/health problems. Shift/night workers should have the opportunity to be screened for disorders linked with their work.

## 1. Introduction

Circadian rhythms regulate the life functions of a human being. Each of us has our own preferences regarding sleep; on this basis, you can determine your chronotype. Evidence shows that chronotype habits are genetically regulated (approximately 45% of chronotype preferences can be explained by gene polymorphism) [1,2]. Despite its economic and social significance, shift work causes disruption of diurnal rhythms and influences the well-being of shift workers [3]. Employees working in shifts are exposed to many threats affecting their health, quality of life and safety at work. Workers who perform their work only at night are particularly vulnerable [4]. Nurses and midwives employed in inpatient care dominate in this group, which is obvious due to the need to provide patients with round-the-clock care. Both in Poland and other countries around the world, numerous studies were conducted to determine the impact of work in shifts, including night shifts, on various aspects related to the functioning of the body. Nurses are the largest group of healthcare providers who are responsible for providing high quality patient care. Sleep problems can lead to distraction and apathy, interfering with work capacity. Long-term sleep problems can lead to serious effects such as delayed thinking, hypomnesia, slow reaction, fatigue, irritability, increased frequency of depression and suicidal thoughts [4,5,6,7,8,9]. Furthermore, shift work may increase the risk of various diseases, such as cancer, diabetes or cardiovascular diseases [10,11,12,13].

### Influence of Shift/Night Work on Health and Sleep Quality

Decades of research have identified many biochemical processes involved in the regulation of sleep homeostasis. Sleep plays an important role in our cognitive abilities and is not simply the opposite state of wakefulness, but rather a dynamic process involving changes in neurotransmitter release, immune response and metabolic status. Sleep disorders impact not only circadian patterns, but also mood, perception, memory, stress response and daily functioning. Performing strenuous work at night affects the quality of sleep [14,15]. There are many factors influencing our sleep, i.e., physiological and environmental changes, emotions and cognitive functioning; thus, sleep quality is a very complex matter. Sleep quality influences an individual’s quality of life and health perception. To our knowledge, shift/night work may cause significant sleep disruptions, low sleep quality and daytime sleepiness [16]. 

In 2019, The International Agency for Research on Cancer (IARC) classified night shift work as probably carcinogenic. It is hypothesised that an altered circadian rhythm can influence the expression of circadian genes. Disruption of the circadian central clock may lead to asynchronous cell proliferation [17]. Night shift work might be associated with breast cancer in women, prostate cancer and chronic lymphocytic leukaemia [18]. 

Recent studies found that night shift work has an impact on female reproductive health and fertility. Night work affects the circadian rhythm and disrupts hormonal balance. The hypothalamic-pituitary-ovarian axis (responsible for follicular maturation and ovulation) is controlled by the circadian clock; therefore, night work may cause abnormal menstrual cycles. There is a link between melatonin levels and maternal circadian rhythm. Women who work night shifts have lower melatonin levels due to light-at-night exposure. Melatonin is primarily synthesised in the pineal gland and is also found in the ovaries or the placenta. Furthermore, it is thought to play a crucial role in the maintenance of full-term pregnancies [19,20].

It was established that night shift work may influence dietary intake and alter melatonin levels, which regulates metabolism-related hormones, i.e., insulin, cortisol and leptin. Prolonged asynchrony of circadian rhythmicity can affect body weight and lead to adiposity. Moreover, the night shift makes workers eat during resting time, which leads to increased diurnal caloric intake [21,22].

Several epidemiological studies have indicated that shift work increases the risk of developing glucose intolerance or type 2 diabetes. The human circadian system is a complex structure. As previously mentioned, melatonin (which plays a crucial role in circadian rhythms) regulates insulin secretion and insulin sensitivity. Decreased melatonin levels are concomitant with inadequate glycaemic control and increased risk of developing type 2 diabetes [23,24,25].

Elevated blood pressure, dyslipidaemia, increased waist circumference and body mass index are known factors of cardiovascular diseases. Several studies observed that night shift work is correlated with cardiometabolic risk. Atherosclerosis and altered drug intake for hypertension were observed [26,27,28]. 

## 2. Methods 

### 2.1. Study Design 

The work uses a systematic review (SR), which aims to search, synthesise and assess existing knowledge on a given topic. 

The aims of this study were to:

▪ establish an association between night/shift work and health.
▪ identify disorders linked to night/shift work.
▪ identify the incidence of sleep disorders in night/shift workers.
▪ evaluate the impact of night/shift work on sleep quality.

### 2.2. Search Methods 

Medline databases were searched: PubMed, Ebsco, OVID, Scopus and Web of Science. The following words were used for verification: nurse, healthcare workers, shift work, night work, sleep, sleep disorders, sleep quality, health, risk. The search was limited to the latest studies published in English and carried out in the years 2019–2024. Single keywords were introduced, as well as a combination of them with AND, OR and both operators. The number of citations obtained during each search attempt was scanned and reduced according to the inclusion criteria. Only studies on healthcare workers working in medical facilities on shifts that assessed sleep quality and health problems were selected. The last search was performed on 12 July 2024. A total of 403 articles were found in the databases corresponding to the discussed issue. After considering the assumed review criteria; finally, 38 articles were included in the analysis. 

### 2.3. Study Selection 

Inclusion criteria

▪ Years of publication: 2019–2024 (research was limited to the latest studies, hence the years of publication).
▪ Publication type: original papers only.
▪ Studies carried out on healthcare workers.

Exclusion criteria

▪ Year of publication earlier than 2019.
▪ Publications type: reviews, meta-analyses, letters, opinions.
▪ Studies carried out on shift/night workers other than healthcare employees.

### 2.4. Research Variables and Strategy 

All papers selected for inclusion in the systematic review were subjected to the appraisal of two independent researchers. The Joanna Briggs Institute (JBI) Critical Appraisal Checklist for Cross-Sectional Studies was used to assess the methodological quality of the study and the possibility of bias in its design [29]. 

## 3. Results 

**Figure 1:**
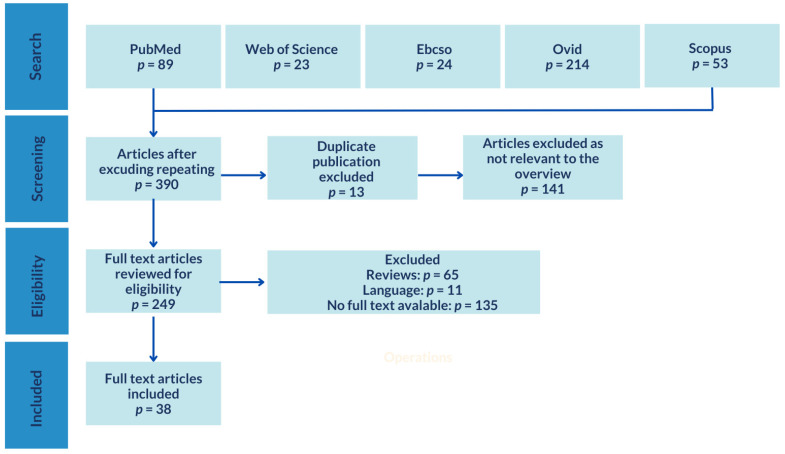
Search strategy for eligible articles.

A total of 38 papers that met the inclusion criteria were selected for final analysis. Seven articles described the influence of shift/night work on overall sleep quality, whereas thirty-one studies referred to the link between shift/night work and various health problems. The search strategy is presented in Figure 1. 

Healthcare workers who participated in the studies were physicians, nurses, midwives and technicians working in various hospitals. Most studies were cross-sectional in design [30,31,32,33,34,35,36,37,38,39,40,41,42,43,44,45,46,47,48,49,50,51,52]. Only one study did not include any control group—a small study by Imes et al. describing the impact of rotating shifts on health [53]—and some researchers did not give a clear definition of night work [31,40,50,54]. Most studies defined night or shift work exposure as work performed between midnight and 5 a.m. [34,35,39,41,42,49,52,55,56,57,58,59,60,61,62]. Moreover, many of the surveyed employees worked 3-shift rotations (morning, evening and night shifts) [30,33,35,48,50,59,60,61,63]. A few researchers defined night work exposure as at least 2–3 night shifts per month for the last 2 years. Most of the studies were carried out on large cohorts, while 11 studies included small sample groups (less than 100 subjects) [31,32,35,49,50,51,53,64,65,66].

Most authors who investigated the quality of sleep amongst shift/night workers used the Pittsburgh Quality Index questionnaire, and some researchers used their own validated questionnaire. Most authors have shown a link between sleep disturbance or its quality and shift work/night work. The most reported sleep disturbances were difficulty falling asleep and waking up at night, short sleep duration and low subjective sleep quality [30,37,38,39,40,46,51].

Concerning health problems, several authors found evidence of the association between shift/night work and various disorders: increased risk of cardiometabolic and cerebrovascular diseases, DNA damage and increased risk of breast cancer, preterm birth and metabolic and immune disruptions [31,32,33,41,42,43,47,48,51,52,55,56,57,63,64,65,66]. The evaluation of the results is described in Table 1. Study characteristics can be found in the Supplementary Materials in Table S1.

## 4. Discussion

Most authors have shown a link between sleep disturbance or its quality and shift work/night work [31,43,46,47,56]; only one study showed no relationship between day or night shifts and the quality of sleep, which was described in line with a previous study by McDowall et al., who concluded that poor sleep quality was present in both shift- and non-shift workers [42,68]. Poor sleep quality was observed in all healthcare workers in a study by Uekata et al.; however, a three-shift schedule was the most significant factor for poorer subjective sleep quality when compared to other work schedules (12.5 or 16 h night shifts) [30]. Most reported sleep disturbances were fragmented sleep, a long sleep latency and short sleep duration [53]. A few studies investigated the level of fatigue during the day [37,46,53,58]. Jaradat et al. found that fatigue was prevalent in more than two-thirds of participants, but it was not investigated whether night shifts were associated with this factor [37]. An earlier study by Gomez-Garcia et al. also revealed a significant level of daytime sleepiness (more than half of respondents), furthermore Roman et al. observed poorer sleep efficiency in night workers, thus fatigue and sleepiness might have been influenced by sleep quality [51,62]. On the other hand, Alshahrani et al. discovered no difference between studied groups (shift work vs. non-shift work) concerning daytime sleepiness; nonetheless, the authors did not ask about naps or drinking caffeinated beverages during shifts. In addition, the research was performed in Saudi Arabia, where the international standards for working time are followed; therefore, the employees must have an appropriate amount of time off from work. This prevents employees from rapid returns and excessive or long working hours [69].

Some authors studied the chronotype of participants to determine the relationship between the type of changes (shifts) and the quality of sleep [30,36,37,41]. Uekata et al. noted that workers with evening chronotype had poor sleep quality on working days, whereas morning chronotype workers experienced poor sleep quality on work-free days [30]. Earlier studies carried out by Lee et al. revealed that nurses with the evening chronotype were more susceptible to emotional disturbances and insomnia [1]. Thus, it can be hypothesised that working shifts following one’s chronotype has a less distressing impact on health and sleep quality, as any sudden changes can be more disturbing than working regular fixed shifts.

Another noteworthy fact is that most of the respondents were women. In addition to professional work, most women also do non-professional work and are burdened with the care of other family members. Some authors asked in their questionnaires about having children or family members dependent on their care (dependents), since the need to combine many occupational and non-occupational duties can contribute to the occurrence of daytime fatigue, shortening of sleep or deterioration of its quality [30,37].

Not only did the studies included in the review show a correlation between shift work and sleep disturbances, but they also found a link between night/ shift work and physical health. Some studies have shown a relationship between night work and the presence of abdominal obesity and metabolic syndrome, which affect the occurrence of coronary heart disease and diabetes [41,47]. Metabolic syndrome is a cluster of disorders that occur simultaneously. These disorders include excess adipose tissue around the waist, high blood pressure, high glucose levels and cholesterol/triglycerides imbalance [70]. Because excess adipose tissue is a result of surplus food intake, it was investigated whether night shift workers were more prone to weight gain. In a few studies, there was a correspondence between night work and increased body weight [50,58,70]. In a previous study by Buchvold et al., night shift work was found to be a far greater risk factor for obesity than working mixed shifts [71]. On the contrary, other studies did not establish the relation of shift work with obesity, which might be a result of some methodological differences [33,72]. Terada et al. established that higher snacking frequency during night shifts was associated with a greater percentage of body fat as well as body mass index and waist circumference. Moreover, snacks consumed by shift-working nurses were mostly unhealthy (chocolate, chips, sweet beverages) [35]. Nonetheless, the researchers did not find any differences between the studied groups regarding metabolic indicators. 

Rollin et al. found that more than half of night shift workers gained weight (approximately 8 kg) during the night work period due to snacking and hyperphagia. As it is difficult not to reach for snacks during night shifts, it can be avoided by eating a full meal beforehand [35,72]. In turn, Park et al. reached interesting conclusions regarding meals after a night shift: the sooner nurses ate a high-protein and high-calorie meal, the better the quality of sleep they achieved [73]. 

Besides weight gain, researchers found that night shift work or rotating shift work was linked with elevated glucose levels/diabetes (or insulin resistance), elevated blood pressure and overall metabolic syndrome [32,41,47]. Multiple studies have shown that insufficient and/or irregular sleep promotes glucose intolerance. Studies investigating women’s health revealed that extended periods of night shift work were linked to a greater risk of developing type 2 diabetes in women [72,73,74,75], which is in line with the latest studies regarding night-working nurses [9,32]. Given the increasing evidence underlying the role of gut microbiota in the development of type 2 diabetes, circadian misalignment could affect microbiota diversity, thus promoting insulin resistance [9]. In line with this evidence, Loef et al. did not find a connection between shift work and metabolic syndrome [33].

It was hypothesised that insulin resistance is correlated with disruption of circadian rhythms and cannot be explained solely by anthropometric factors (BMI, WC)/body fat content and was confirmed in a previous study by Hansen et al.; the risk for diabetes was pertinent to all women working night shifts [76,77]. Moreover, Kiranmala et al. linked insulin resistance to altered lipid levels, which suggests that postprandial triglyceride levels play an important role in insulin resistance mechanisms [32]. Worth mentioning is another fact: night shift workers had short periods of fasting time (due to snacking during shifts), which can be a complementary risk factor for insulin resistance. 

Another study investigating the association between night work and dyslipidaemia included South Korean workers, in which it was established that men were more susceptible to altered lipid levels [26]. The latest studies showed a correlation between night/shift work to some metabolic changes: van den Langenberg et al. connected shift work with impaired lipid metabolism. Furthermore, Borroni et al. demonstrated altered levels of some metabolites (glycerophospholipids, sphingolipids, serotonin, taurine, aspartic acid) [51,52]. However, there are some limitations to the aforementioned studies; most of them were conducted on a small study group and were cross-sectional in their design.

Most research regarding night/shift work in healthcare is focused on nurses, whose vast majority are women, thus the research produces somewhat greater bias (as there are not enough male participants to compare).

Several studies have shown a correlation between night/shift work and elevated blood pressure. Nascimento et al. concluded that fixed night shift work was related to altered blood pressure during sleep in ambulatory blood pressure monitoring (ABPM), together with Solymanzadeh et al. and Torun et al., whose studies revealed a connection between rotational shift work and hypertension [34,45,65]. These results are in place with another study describing hypertension risk in petrochemical plant shift workers [78]. Hypertension is the most prevalent risk factor for strokes [79]; therefore, night work, which alters blood pressure, may be a risk for cerebrovascular incidences. This hypothesis was confirmed in a large cohort of 30,460 employees by Bigert et al. [63]. 

Because most of the nursing staff are women, healthcare workers must be familiar with threats related to night/shift work in terms of women’s health. Some studies suggested that demanding and physically intense jobs increase the risk for preterm delivery, miscarriage, low birth weight birth weight, hypertension and preeclampsia, especially when it comes to night shift work [80,81], which is in line with the studied research of this review [55]. On the other hand, Specht et al. did not find night work itself to be a risk factor for preterm birth. Noteworthy is the fact that they found switching from night shifts in the first trimester to day work only during the second trimester was related to an increased risk of preterm birth. It might indicate that not only night work, but any hormonal imbalance may be a risk factor for carrying the pregnancy to term [19]. Another interesting study was conducted by Nehme et al., in which the researchers measured melatonin levels in pregnant women and compared the health status of the newborns. The results indicated that night workers had assisted deliveries and the newborns had lower Apgar scores and trouble breastfeeding [18]. Unfortunately, the study was limited to its numbers—only three women were eligible for the study, so there is no possibility of generalising their results to a larger population. 

Furthermore, there is some evidence of a link between night work and breast cancer. Carugno et al. established that there is a link between prolonged exposure to night shifts and molecular changes that may be involved in processes such as the ageing of cells and genome instability, especially TP53 and BRCA1, which encode tumour suppressors. It is in line with another study by Fagundo-Rivera et al., in which not working in shifts or at night was linked with a reduced risk of breast cancer [42,64]. These studies are in accordance with other research, in which night work was found to be damaging to DNA; in participants working overnight, DNA hypermethylation and breaks were increased [31,49,82]. Ahmadi et al. studied the influence of night shifts on DNA methylation of circadian genes, and it appeared that there was a positive correlation. Interestingly, in a study by Lahtinen et al., a break from night work led to changes in DNA methylation, which influenced the activity of the NMDA receptor, which could mean that the risk of breast cancer decreases after the night work is stopped [83]. However, the risk of breast cancer increases with age, so it is not possible to simply hypothesize about the incidence of breast cancer after retiring. On the contrary, Heckmann et al. found that night work was not associated with skin cancer, which is probably due to reduced exposure to sunlight [84].

Only a few studies have explored the possibility of the impact of night/shift work on the immune system. Loef et al. established that shift workers were prone to more severe acute respiratory infections when compared to non-shift workers, whereas Faraut et al. noted that night work may decrease vaccination efficiency due to altered immune patterns (different blood concentrations of total lymphocytes and T-helpers lymphocytes when compared to day shifters) [48,67].

One study described the influence of night work on the incidence of psychiatric disturbances—authors noted that working night shifts for a long period (6 years or more) was associated with mood and neurotic disorders [59]. These findings are consistent with other studies that have shown a correlation between night work and bipolar disorder, depression and neurotic behaviours [85,86,87]. Even in the medical community, the stigma associated with mental health conditions impacts willingness to seek help or disclose a mental health problem, which can result in ostracization from co-workers and an increased risk of suicide. This is also a reason for self-treatment and not seeking help from other medical professionals [88].

## 5. Implications for Practice

- All staff members should be aware of the risks related to night/shift work.
- Night work should be a personal choice.
- If possible, staff members should be able to switch to day shifts when they feel that night shifts are causing any distress.
- Screening for disorders linked to night/shift work should be done regularly.
- If possible, the work schedule should be adjusted to individual chronotypes.
- Shift/night workers should be able to consult with a sleep specialist when they feel their job interferes with daytime fatigue.
- Shift-working healthcare professionals may require a more specific dietary program to improve their health.

## 6. Conclusions

Limitations of this study include the study design; the self-report method (night work exposure) might have influenced the results. Some studies were too small to objectively generalise the results on the whole population. Nevertheless, the research results clearly show a significant impact of night work on sleep quality, but also on health in general. 

Healthcare workers should be aware of the risks associated with night work to take measures to prevent sleep/health problems. Shift/night workers should have the opportunity to be screened for disorders linked with their work.

## Figures and Tables

**Table 1:** PICOS (Population, Investigated condition, Control group, Outcome, Study design) evaluation.

Author/Date	Population	Investigated Condition	Comparison/Control Group	Outcome	Study Design
Imes et al., 2019 [53]	Nurses	Impact of rotating shifts on health	No control group	Shift work was associated with sleep-related impairment, fatigue, emotional distress (anger), and worse memory and concentration	A within-subject study
Uekata et al., 2019 [30]	Nurses, midwives	Impact of rotating shift schedule on sleep quality	Day-only workers vs. rotating shift workers	Poor sleep quality was high among all nurses and midwives, especially those engaged in three-shift rotations	A cross-sectional study
Carugno et al., 2019 [64]	Nurses	Association between night work and DNA methylation of tumour suppressor	Night shift workers vs. non-shift workers	There was an association between night shift work and DNA alterations potentially related to a higher carcinogenic risk	A cohort study
Begtrup et al., 2019 [55]	Hospital employees: nurses, physicians, midwives, nurses’ assistants, other	Influence of night work on miscarriage	Night shift workers vs. no-night+ workers	There was an increased risk of miscarriage among women who had night work the previous week, and among women with cumulated numbers of night shifts	A cohort study
Cheung et al., 2019 [31]	Physicians	Influence of night work on DNA disruption	Night shift workers vs. no-night workers	Acute sleep deprivation and a frequently disrupted sleep cycle were associated with DNA damage	A cross-sectional observational study
Kiranmala et al., 2019 [32]	Nurses, physicians, other healthcare workers	Influence of rotational shift work on postprandial triglyceride levels and insulin resistance	Non-shift workers vs. shift workers	Rotational night shift work might negatively impact on metabolic parameters	A cross-sectional study
Loef et al., 2019 [33]	Healthcare workers (75% nurses, 25% other)	Influence of shift work on metabolic risk factors	Non-shift workers vs. shift workers	No evidence was observed that could underlie a link between shift work and cardiometabolic diseases	A cross-sectional study
Loef et al., 2019 [56]	Healthcare workers (75% nurses, 25% other)	Influence of shift work on respiratory infections	Non-shift workers vs. shift workers	Shift workers had more acute respiratory illnesses and more severe symptoms than non-shift workers	A prospective cohort study
Loef et al., 2019 [67]	Healthcare workers (74% nurses, 26% other)	Immunological effects of shift work	Night shift workers vs. non-shift workers	Chronic exposure to night shift work as well as recent night shift work may influence workers’ immune status	A prospective cohort study
Nascimento et al., 2019 [34]	Nurses (44.2%), nursing assistants (55.8)	Influence of shift work on blood pressure, the presence of burnout and common mental disorders	Shift workers vs. non-shift workers	Shift work was associated with a higher prevalence of work-related inadequate habits and lifestyles and altered (elevated) sleep blood pressure	A cross-sectional study
Terada et al., 2019 [35]	Nurses	Association between shift work, dietary habits and mood disturbance	Non-shift workers vs. shift workers	Shift-working nurses exhibited shorter fasting duration, larger diurnal energy intake and higher total mood disturbance score	A cross-sectional study
Rizza et al., 2019 [57]	Healthcare workers	Association between shift work and occurrence of thyroid nodules	Day workers vs. rotating night shift workers	Alteration in the molecular clocks typical of rotating night shift workers harbours a higher risk of thyroid nodule development	A retrospective cohort study
Bani Issa et al., 2020 [36]	Nurses	Influence of rotating night shift work on sleep quality	Fixed day workers vs. rotating night shift workers	Rotating night shift nurses had better sleep than working fixed day shifts. Proper shift assignment and chronotype alignment with shift work were related to better sleep quality.	A cross-sectional study
Jaradat et al., 2020 [37]	Physicians	Sleep quality and health-related problems of shift work	On-call shift workers vs. no on-call shift workers	The majority (90%) reported poor sleep quality; residents having six on-calls or more per month had significantly poorer sleep quality, as well as higher anxiety and depression scores compared to their counterparts	A cross-sectional study
Ljevak et al., 2020 [38]	Nurses	Influence of shift work on psychological functioning and quality of life	Rotating shift workers (51%) vs. day workers (49%)	Increased anxiety, stress, psychosomatic symptoms and sleep disturbances were more common in shift workers	A cross-sectional study
Brum et al., 2020 [58]	Hospital workers (direct patient care, administration support, maintenance)	Association between night shift work and obesity	Day vs. night workers	Night work was a determining risk factor for abdominal obesity	A cross-sectional study
Feng et al., 2021 [39]	Nurses	Association between night shift and sleep quality and health	Day shift vs. night shift workers	Night shift work was significantly associated with poor sleep quality and poor health	A cross-sectional study
Qanash et al., 2021 [40]	Nurses, physicians, other healthcare providers	Impact of night shifts on sleeping patterns, psychosocial and physical well-being	Night shift workers (272) vs. day workers (80)	Night shift workers were more likely to have sleep disturbances, particularly in terms of initiating sleep, staying asleep and duration of sleep compared with day shift workers	A cross-sectional study
Aslam et al., 2021 [65]	Nurses, physicians, other healthcare personnel	Effects of rotational night shift work on expression of circadian genes and its association with postprandial triglyceride levels	Night shift workers vs. no-night shift workers	The study showed altered expression of several circadian genes with postprandial triglyceride and insulin resistance parameters in rotational night shift workers	A cross-sectional study
Cheng et al., 2021 [41]	Nurses, medical technicians, administrative clerks, pharmacists	Influence of night shift work on the risk of metabolic syndrome	Night workers vs. day workers	Night shift work was associated with metabolic risk factors	A retrospective cohort study
Fagundo-Rivera et al., 2021 [42]	Nurses	A risk of breast cancer in shift workers	Nurses diagnosed with breast cancer vs. cancer-free controls	The estimated risk for breast cancer was higher amongst night shifters who had more than 3 nights/ month and worked more than 16 years	A cross-sectional study
Jordakieva et al., 2021 [43]	Hospital employees (nursing staff, administrative personnel)	Quality of life and cardiovascular risk markers in ageing night shift workers	Night vs. day workers	Quality of life and cardiovascular markers did not significantly differ between rotating night shift and day workers	A cross-sectional study
Jørgensen et al., 2021 [59]	Nurses	Shift work and incidence of psychiatric disorders	Shift (37.8%) vs. day workers (62.2%)	Night shift work was associated with an increased risk of mood and neurotic disorders, as well as substance use	A retrospective cohort study
Ljevak et al., 2021 [44]	Nurses	Influence of shift work on overall health status	Rotating shift workers (51%) vs. day workers (49%)	Shift employees are significantly more burdened with appetite loss, nausea, heartburn and weight gain. Differences in the severity of cardiovascular disorders were not statistically significant between the two groups	A comparative cross-sectional study
Kader et al., 2021 [60]	Nurses, nursing assistants	Night and shift work characteristics and incident ischemic heart disease and atrial fibrillation among healthcare employees	Rotating night or night shift workers vs. no-night workers	An excess risk of incident ischaemic heart disease among employees who during the preceding year worked permanent night shifts, compared to permanent day workers	A prospective cohort study
Solymanzadeh et al., 2021 [45]	Nurses	A relationship between rotating shift work and blood pressure	Rotating shift workers (50%) vs. no-night workers (50%)	Rotating shift work was associated with a greater increase in BMI and increased risk of high BP compared to day workers	A cross-sectional study
Chang et al., 2022 [46]	Nurses	Influence of shift work on sleep quality and fatigue	Day shift (42.5%) vs. shift workers (57.5%)	Shift workers had poorer sleep quality, which was found to impact their fatigue	A cross-sectional study
Ahmadi et al., 2022 [66]	Hospital employees	DNA methylation of circadian genes and markers of cardiometabolic risk	Day shift (51%) vs. night shift workers (49%)	Night shift work influenced DNA methylation of circadian genes, which may contribute to increased cardiometabolic risk	A cross-sectional study
Bahinipati et al., 2022 [47]	Hospital employees	Effect of night shifts on the development of metabolic syndrome	Day shift (44%) vs. night shift workers (56%)	Night shift work is associated with an increase in pro-inflammatory markers and the development of risk factors leading to metabolic syndrome	A cross-sectional study
Bigert et al., 2022 [63]	Nurses, nursing assistants	Night and shift work and incidence of cerebrovascular disease	Shift or night workers vs. no-night workers	Night shift work was associated with a higher risk of cerebrovascular disease	A prospective cohort study
Faraut et al., 2022 [48]	Nurses, nursing assistants	Effects of circadian and sleep disruptions on immune biomarkers among rotating night shift healthcare workers	Permanent night shifters vs. day shifters rotating between morning and afternoon shifts	Disrupted pattern expression of immune cells in hospital night workers could increase vulnerability to infections and reduce vaccination efficiency in night workers	A cross-sectional study
Ritonja et al., 2022 [49]	Nurses	Relationship between melatonin secretion patterns and circadian gene methylation among day and night shift workers	Fixed day shifters vs. night shifters	Melatonin was associated with changes in DNA methylation of circadian genes among night shift workers	A cross-sectional study
Sooriyaarachchi et al., 2022 [50]	Nurses, patient care assistants, administrative personnel, security personnel	Comparison of the body composition parameters between shift workers and non-shift workers	Day workers vs. shift workers	Prolonged exposure to shift work was associated with a higher body fat percentage. BMI and waist circumference were significantly higher among shift-working women	A cross-sectional study
Borroni et al., 2023 [51]	Nurses	Comparison of metabolomic profiles between night shift and non-night shift workers	Night workers vs. no-night workers	The study shows altered levels of some metabolites in night shift workers	A cross-sectional study
Roman et al., 2023 [54]	Nurses	Association between rotating night shifts and health	Fixed shift workers vs. rotating shift workers	Nurses who worked rotating shifts more often reported poor sleep efficiency, abdominal pain and anxiety.	An observational, comparative study
Van den Langenberg et al., 2023 [52]	Female Nurses and Paramedic Staff	Impact of shift work on metabolic biomarkers	Night shift vs. day shift workers	Night shifts were associated with unfavourable patterns in fatty acid profiles	A cross-sectional study
Viklund et al., 2023 [61]	Nurses and Nursing assistants	Effects of night and shift work on type 2 diabetes and hypertension	Night shift vs. mixed shift vs. day workers	The night shift was associated with an increased risk of type 2 diabetes but not hypertension	A longitudinal study
Torun et al., 2024 [62]	Physicians, nurses and other healthcare workers	Effect of night work on blood pressure	Night shift vs. no-night shift workers	Statistically significant differences were noted between the groups in the daylight-night ratios of systolic and diastolic pressures	An observational, comparative study

## Supplementary Materials

The following supporting information can be downloaded at: https://www.mdpi.com/article/10.3390/jcm13154505/s1, Table S1. Study characteristics.
