# Supplementary material for: The Impact of Night Work on the Sleep and Health of Medical Staff—A Review of the Latest Scientific Reports
Source: J Clin Med. 2024 Aug 1;13(15):4505. doi: 10.3390/jcm13154505 (PMC11313391; doi:10.3390/jcm13154505)
Supplement: Supplementary file 1 [file jcm-13-04505-s001.zip › jcm-3096239-supplementary.pdf]

Supplementary Materials: Table S1. Study characteristics.

Author/Date	Population	Age	Place of employment/ country	Shift/night work exposure/definition
**Imes et al., 2019** [53]	23 nurses; 21 females, 2 males	Mean age: 27.0 ± 4.5	Hospital (Intensive Care Unit), USA	Rotating shift work was defined as at least three consecutive day shifts followed by at least three consecutive night shifts
**Uekata et al., 2019** [30]	1253 female nurses and midwives	Age range: 25–52 years old	Hospitals, Japan	Rotating shifts included 12,5h night shifts, 16h night shifts and three shift rotations
**Carugno et al., 2019** [64]	97 female nurses	Mean age: 35.9 ±5.4 years old	Hospital, Italy	Night shift workers were defined as employees who had been working in shifts for at least 2 years; no clear definition of night work
**Begtrup et al., 2019** [55]	22774 female employees	Mean age: 30.5 ±3.9 for exposed to night shifts employees 30.9 ± 4,4 for non-exposed	Public hospitals, Denmark	A night shift was defined as at least 3h between midnight and 5 a.m. Exposed employees were defined as participants with ≥ 1 night shifts between 3 and 21 pregnancy weeks
**Cheung et al., 2019** [31]	49 participants (24 males, 25 females)	Mean age: on-site call participants: 28.04 ±2.4; no-night shifts participants: 33.88 ±7.8	Two hospitals, Hong Kong	No clear definition of night work. Participants worked 'overnight on-site calls'
**Kiranmala et al., 2019** [32]	40 participants (26 males, 14 females)	Mean age: non-shift workers: 29.7 ± 2.2; shift workers: 29.7 ± 3.9	Hospital, India	Night work was defined as ≥ 4 nights duties per month at least for the past 1 year
**Loef et al., 2019** [33]	596 healthcare workers (521 females, 75 males)	Mean age: shift workers 40.9 ±12.2, non-shift workers 46.8 ± 11.2	Six hospitals, Netherlands	Shift work was defined as rotating between day (7.30 a.m.–4 p.m.), evening (3–11 p.m.) and night shifts (11 p.m.–7.45 a.m. or 0–6 a.m.) for at least 6 months prior to the study

**Loef et al., 2019** [56]	589 healthcare workers (87% females, 13% males)	Mean age: non–shift workers 40.9 years, shift workers 46.3 years	Six hospitals, Netherlands	Shift work was defined as rotating between day (7.30 a.m.–4 p.m.), evening (3–11 p.m.) and night shifts (11 p.m.–7.45 a.m. or 0–6 a.m.) for at least 6 months prior to the study
**Loef et al., 2019** [67]	311 healthcare workers (88% females, 12% males)	Mean age: non–shift workers 47.4 ± 9.9 nightNight -shift workers 42.1 ± 11.9	Hospitals, Netherlands	Night -shift workers worked rotating shifts including night shifts (0–6 a.m.).
**Nascimento et al., 2019** [34]	231 nurses (138 professionals working in shifts)	Mean age: 39.6 years	Public hospital, Brazil	Shift work was defined as work performed intermittently and at different times, without a fixed schedule. Non-shift workers worked fixed day or night shifts
**Terada et al., 2019** [35]	73 female nurses	Mean age: non-shift workers 50.7 ± 7.8; shift workers 41.3 ± 11.9	Hospital, Canada	Shift work was defined as work outside of daytime hours including irregular or rotating schedules, evening and night work
**Rizza et al., 2019** [57]	299 healthcare workers (70% females, 30% males); nurses (84), physicians (96), technicians (88), other (31)	Mean age: rotating night -shift workers 38.7 ± 9.4; day workers 36.5 ± 7.9 years	Hospital, Italy	Night work was defined as work between midnight and 6 a.m. Rotating night -shift schedule included 4–7 12-h-night shifts/month
**Bani Issa et al., 2020** [36]	520 female nurses	Mean age: 36.7 ± 8.5 years	Hospitals and primary care centres, UAE	Rotating night shift was defined as having night, day and evening shifts in the same month
**Jaradat et al., 2020** [37]	122 male, 79 female physicians	Mean age: 27 years	Hospital, Jordan	The on-call shift started at 4 p.m. at the end of a normal working day and lasted until 8 a.m. the next day
**Ljevak et al., 2020** [38]	157 hospital nursing professionals: 135 female and 22 male employees	Mean age: 33.3 years	Hospital, Bosnia and Hercegovina	Shift work included obligatory night shifts. Shift nurses worked in fixed schedules: 12-h day

				shift / 24 h off / 12-h night shift / 48 h off
**Brum et al., 2020** [58]	139 women and 61 men	Mean age: 43.2 ± 9.3	Hospital, Brazil	Day shift was from 6 to 8 h a day (between 7 a.m. and 7 p.m.), night working hours were from 6 to 12 h (6 p.m. till midnight or 7 p.m.–7 a.m.)
**Feng et al., 2021** [39]	3206 nurses (95,98% females)	Mean age: 33.1 ± 6.3	Hospital, China	Night shift nurses worked at least 8 h between 5 p.m.–8 a.m.; day shift nurses worked at least 8 h from 8 a.m.–11 p.m.
**Qanash et al., 2021** [40]	352 healthcare providers (Male 174, female 178)	Mean age: 30.6 ± 7.52	Tertiary hospitals, Saudi Arabia	No clear definition
**Aslam et al., 2021** [65]	40 healthcare workers ((26 males, 14 females)	Mean age: no-night shift workers 29.7 ±2.23, night shift workers 29.7 ± 3.92 years	Hospital, India	Rotational night shift work rotational was defined as ≥ 4 night shifts/month for a minimum of 1 year (napping was not allowed during the night shift duties)
**Cheng et al., 2021** [41]	5775 nurses (56.9%), medical technicians (22.2%), administrative clerks (16.2%), pharmacists (4.8%)	Mean age: day workers 32.9 ± 8.7, night workers 27.6 ± 5.1	Hospitals, Taiwan	Night shifts were defined as ≥ 3 working hours between midnight and 5 a.m.
**Fagundo-Rivera et al., 2021** [42]	558 nurses	Mean age 41 years old	Public/private healthcare centres, Spain	Night work was defined as working at least 3 h between midnight and 5 a.m. 19.2% of the sample worked on permanent shifts (mostly mornings) and 74.5% worked on rotating shifts; 27.1% worked on night shifts only
**Jordakieva et al., 2021** [43]	70 participants:<br>51 nurses,<br>1 nursing assistant,<br>14 administrative personnel (91.4% females)	Mean age: 52 ± 4 years old	Hospital, Austria	Night -workers were defined as employees working a rotating shift system with > 3 night shifts/month and controls worked permanent day schedules
**Jørgensen et al., 2021** [59]	19 964 female nurses	Mean age: 50.4 ± 4.9	Data collected via National Health	Typically, day shift was from 7 a.m. to 3

			Service Register, Denmark	p.m., evening shift from 3 p.m. to 11 p.m., night shift from 11 p.m. to 7 a.m. Rotating shift refers to rotation between at least two of the following: day, evening and night work
**Ljevak et al., 2021** [44]	157 hospital nursing professionals: 135 female and 22 male employees	Mean age: 33.3 ± 8 years	Hospital, Croatia	Rotating shifts included 12-h day shift / 24 h off, followed by 12-h night shift / 48 h off
**Kader et al., 2021** [60]	30 398 healthcare employees (87,6% females	Age range: ≤ 40 years old–51% 41–50 years old–25% > 50 years old—24%	In- or outpatient care, Sweden	Employees worked the following shifts: day (6 a.m.–6 p.m.); afternoon (starts after noon and ends later than 6 p.m.); night (≥ 3 h of work between 10 p.m. and 6 a.m.)
**Solymanzadeh et al., 2021** [45]	120 nurses (62,5% females)	Mean age: rotating shift workers 32.20 ± 2.97; no-night shift workers 37.43 ± 2.90 years old	Hospital, Iran	Rotating schedule included: morning shift (from 7:30 a.m. to 1:30 p.m.), evening shift (1:30 p.m. to 7:30 p.m.), and night shift (7:30 p.m. to 07:30 a.m.). shift workers had 2 morning shifts, 3 evening shifts, and 1 night shift with 1 day off per week.
**Chang et al., 2022** [46]	181 female clinical nurses	Mean age: 26.19 ± 4.58	Hospital, Taiwan	Day workers had shifts from 8 a.m. to 4 p.m. for the past six months; shift workers had shifts from 4 p.m. to 8 a.m. at least four times per month for the past six months
**Ahmadi et al., 2022** [66]	74 female hospital personnel (nurses, administrative personnel, technicians)	Mean age: day workers 47.74 ± 8.3; night workers 44.28 ± 10.5	Hospital, Canada	A rotating schedule typically included two 12-h days, two 12-h nights and five days off. Day workers had a fixed schedule and worked 5 consecutive 8-h

				shifts starting from 8 or 9 a.m.
**Bahinipati et al., 2022** [47]	176 female and 126 male healthcare workers; physicians (57), nurses (98), technicians (33), attendants (51), students (38)	Mean age: 28.6 ± 7.8 years	Hospital, India	Night work was defined as at least 2 night shifts/week or 5/month for at least 2 years
**Bigert et al., 2022** [63]	26 667 women and 3793 men	Age range:<br>≤ 40 years old—50.9%<br>41–50 years old—24.7%<br>> 50 years old—24.4%	Stockholm district (no detailed information), Sweden	Cohort subjects were classified as: day workers (work between 6 a.m. and 6 p.m.), afternoon workers (work between noon and 6 p.m.), night workers (≥3 h between 10 p.m.–6 a.m.)
**Faraut et al., 2022** [48]	191 participants; 93.5% women	Mean age:<br>night workers: 43.5 ± 10.5<br>Morning workers: 38.6 ± 11.1<br>evening workers: 35.7 ± 10.3 years	Hospital, France	Rotating day shifts were either in the morning (7 a.m.–2 p.m.) or in the afternoon (2 p.m.–9 p.m.); permanent night shifters worked between 9 p.m.–7a.m.
**Ritonja et al., 2022** [49]	74 female healthcare employees–38 day workers, 36 night shift workers	Mean age:<br>day workers: 40.9 ± 8.2<br>night workers: 37.5 ± 10.2 years	Hospital, Canada	Night work was defined as night shifts with at least three working hours between midnight and 5 a.m. Typical night shift was between 7 p.m. and 7 a.m.; typical day schedule included 5 consecutive 8-h shifts (starting at 8 or 9 a.m.)
**Sooriyaarachchi et al., 2022** [50]	78 healthcare workers (48.7% males; 51.3% females); 37 day workers, 41 shift workers	Mean age:<br>day workers 36.76 ± 10.78<br>shift workers: 39.17 ± 11.85 years	Hospital, Sri Lanka	No clear definition of night work
**Borroni et al., 2023** [51]	97 female nurses (46 working night shifts)	Mean age:<br>night shift workers 35.1<br>No-night workers 36.8	Hospital, Italy	Night work was performed between 10 p.m. and 6 a.m. or between 9 p.m. and 7 a.m.
**Roman et al., 2023** [54]	380 professionals (353 females), 221 worked in rotating<br>shifts and 159 in fixed shifts	Mean age of fixed shift workers: 24.8 (6.9)<br>Rotating shift workers: 28.0 (6.7)	Hospital, Spain	No clear definition of rotating and fixed shifts
**Van den Langenberg et al., 2023** [52]	237 females	Mean age:	Hospital, Netherlands	Night shift

	(94 day workers, 143 night workers)	night shift workers 43±13<br>Day workers: 39±12		was defined as work that covers ≥1 h between midnight and 6 a.m.
**Viklund et al., 2023** [61]	28 481 participants (25 065 females; 7334 worked only night shifts)	No detailed information	In- and outpatient services, Sweden	Night work was defined as least 3 h between 10 p.m. and 6 a.m.
**Torun et al., 2024** [62]	114 participants (101 females; 48 night workers)	Day workers: 31.3 ± 7.2 night shift workers	Turkey (no detailed information)	Night work was defined as working at least 6 nights per month
